# Thromboelastometric Profile of Sepsis-Induced Coagulopathy and Overt Disseminated Intravascular Coagulation: A Retrospective Cohort Study

**DOI:** 10.3390/jcm15176773

**Published:** 2026-08-31

**Authors:** Piotr F. Czempik

**Affiliations:** 1 Department of Patient Blood Management, Department of Anesthesiology and Intensive Care, Faculty of Medical Sciences in Katowice, Medical University of Silesia, Medyków 14, 40-752 Katowice, Poland; pczempik@sum.edu.pl; 2 Transfusion Committee, University Clinical Center of Medical University of Silesia in Katowice, Medyków 14, 40-752 Katowice, Poland

**Keywords:** clot firmness, disseminated intravascular coagulation, fibrinolysis, mortality, rotational thromboelastometry, sepsis-induced coagulopathy

## Abstract

**Background/Objectives**: Sepsis may cause hemostatic disturbances. Viscoelastic hemostatic assays, such as rotational thromboelastometry (ROTEM), may reveal functional hemostatic changes not captured by conventional tests. The study aimed to analyze ROTEM parameters in sepsis-induced coagulopathy (SIC) and overt disseminated intravascular coagulation (DIC). **Methods**: A retrospective cohort analysis of adult ICU patients with sepsis or septic shock (May 2023–August 2026) who had ROTEM testing (minimum EXTEM and FIBTEM) performed was conducted. Clinical scores (SOFA, ISTH SIC, ISTH overt DIC), conventional coagulation tests, inflammatory biomarkers, and full ROTEM panels (INTEM, EXTEM, FIBTEM, APTEM) were retrived. Group comparisons used Kruskal–Wallis and pairwise Wilcoxon tests; associations with ICU mortality were assessed by multivariable logistic regression adjusted for age, sex, and SOFA. **Results**: Of 167 patients, 40 (23.9%) met ISTH SIC criteria, 24 (14.4%) met overt DIC criteria, and 103 (61.7%) had no SIC/DIC. Patients with SIC or DIC demonstrated slower clot propagation, lower early clot amplitudes and maximal clot firmness, and lower fibrinolytic activity compared to septic patients without coagulopathy. The only ROTEM parameter that differed between SIC and overt DIC was platelet contribution to clot strength, which was lower in DIC compared with the SIC subgroup. In multivariable logistic regression sensitivity analysis, with no SOFA score included as a covariate, no pre-specified ROTEM parameter was associated with ICU mortality after adjustment for age and sex in the current cohort. The coagulopathy subgroup itself was not an independent predictor of ICU death after adjustment. **Conclusions**: Rotational thromboelastometry shows distinct features in patients with SIC and sepsis-induced overt DIC compared with patients without coagulopathy. In the current cohort, the only ROTEM parameter that distinguished overt DIC from SIC was PLT contribution to clot strength. No ROTEM parameter was associated with short-term mortality in the presented cohort.

## 1. Introduction

Sepsis is a leading cause of critical illness worldwide, frequently complicated by profound and dynamic disturbances of hemostasis that contribute to organ dysfunction and mortality. In 2017, there were 48.9 million cases and 11 million deaths due to sepsis globally, which constitutes 19.7% of all deaths [1]. Sepsis-induced coagulopathy (SIC) and sepsis-induced overt disseminated intravascular coagulation (DIC) lie along a continuum of coagulation derangement, ranging from early, often reversible alterations in platelet function and thrombin generation (SIC) to overt consumptive coagulopathy with microvascular thrombosis and bleeding (overt DIC) [2]. Standard laboratory assays—prothrombin time (PT), activated partial thromboplastin time (aPTT), platelet count (PLT), and fibrinogen—offer important but fragmented information and may fail to capture the temporal evolution and global functional status of clot formation and breakdown [3].

Viscoelastic hemostatic assays (VHAs), such as rotational thromboelastometry (ROTEM), provide a real-time, integrative assessment of clot initiation, kinetics, strength, and fibrinolysis, enabling detection of hypercoagulable and hypocoagulable states that are not apparent in standard laboratory tests. Viscoelastic hemostatic assays detect coagulopathy more accurately and earlier, as shown in acute traumatic coagulopathy [4]. Emerging evidence suggests that pathophysiological features of sepsis and septic shock produce certain thromboelastometric changes, some of which may have prognostic and therapeutic implications [5,6,7]. However, the thromboelastometric profiles of SIC and sepsis-induced overt DIC remain incompletely defined.

In this retrospective cohort study, ROTEM parameters from a well-characterized population of patients with sepsis or septic shock were analyzed. The objectives of the study were to analyze ROTEM parameters across the spectrum of sepsis-associated coagulopathy, compare thromboelastometric profiles between patients who met the International Society on Thrombosis and Hemostasis (ISTH) criteria for SIC and overt DIC, and explore associations between thromboelastometric parameters and short-term mortality.

## 2. Materials and Methods

The presented retrospective cohort study analyzed patients diagnosed with sepsis or septic shock, hospitalized in the intensive care unit (ICU) at a large university clinical center, during the time period between May 2023 and August 2026. In the local ICU, ROTEM (ROTEM Delta, Tem Innovations GMBH, Munich, Germany) has been used routinely, among other indications, to assess initial hemostasis in septic patients since the beginning of 2023. Because ROTEM is routinely used in the local ICU to evaluate hemostasis in patients with sepsis or septic shock at the time of admission or shortly thereafter, it can be reasonably assumed that ROTEM was performed in consecutive septic patients the moment they developed acute organ dysfunction warranting ICU admission.

Inclusion criteria were as follows: diagnosis of sepsis or septic shock according to the most recent international consensus [8], and availability of ROTEM results with a minimum of assays assessing the extrinsic coagulation pathway (EXTEM) and fibrinogen/factor XIII (FIBTEM) function.

Exclusion criteria were factors that could affect ROTEM results: liver dysfunction (diagnosed as serum total bilirubin > 2 mg dL^−1^), therapeutic anticoagulation, administration of coagulation factor concentrates, transfusion of pro-hemostatic blood components (cryoprecipitate, fresh frozen plasma, platelet concentrate), surgical or medical bleeding, pregnancy, and inclusion in another research project [5].

The retrieved general data included sex, age, height, weight, and body mass index (BMI). The clinical data retrieved included the presence of sepsis or septic shock; administration of antiplatelet agents and/or anti-thrombotic medications (type and dose); the time interval between the last dose of anti-thrombotic medication and blood withdrawal for ROTEM determination; anatomical source of infection; ICU mortality; and hospital mortality. Sequential Organ Failure Assessment (SOFA) [9], ISTH SIC [10], and ISTH overt DIC [11] scores were calculated. The ISTH SIC score was calculated using the SOFA scoring system without the platelet component—a minimum score of 4 indicated the presence of SIC. A minimum score of 5 on the ISTH overt DIC score indicated the presence of DIC. Sepsis-induced coagulopathy and overt DIC were assigned hierarchically and mutually exclusively following the ISTH two-step diagnostic algorithm: first, all patients were evaluated for ISTH SIC using the SOFA-based scoring system (excluding the platelet component, as recommended), then patients meeting SIC criteria were further evaluated for ISTH overt DIC using the ISTH overt DIC score [12]. The recommended thresholds for moderate and severe D-dimers (DD) were used to calculate the ISTH overt DIC score, namely with moderate elevation corresponding to values exceeding three-fold the upper limit of normal (ULN), and severe elevation corresponding to values exceeding seven-fold the ULN [13]. Biochemical parameters included interleukin 6 (IL-6), procalcitonin (PCT), C-reactive protein (CRP), creatinine, estimated glomerular filtration rate according to the Modification of Diet in Renal Disease (MDRD) formula, blood urea nitrogen, urea, bilirubin, aspartate aminotransferase, alanine aminotransferase, and platelet count (PLT). In the local ICU, every time ROTEM is performed, conventional coagulation tests (CCTs) are determined simultaneously from the same blood sample. The retrieved CCTs included the following: fibrinogen concentration (Clauss method), PT, prothrombin activity, international normalized ratio (INR), aPTT, thrombin time (TT), and DD. The complete ROTEM analysis includes tests assessing the intrinsic coagulation pathway (INTEM), EXTEM, FIBTEM, and fibrinolysis (APTEM). The retrieved ROTEM parameters were as follows: clotting time (CT); clot formation time (CFT); alpha angle (AA); clot amplitude at 5 (A5), 10 (A10), and 20 (A20) minutes; maximal clot firmness (MCF); maximal lysis (ML); and lysis index at 30 (LI30) and 45 (LI45) minutes. Platelet contribution to clot strength was calculated as the difference in MCF between EXTEM and FIBTEM.

Statistical analyses were performed using a licensed statistical software (Stata 18.0 BE, StataCorp LLC, College Station, TX, USA). Normality of continuous variables was assessed using the Shapiro–Wilk test. Continuous variables were presented as the mean with standard deviation (SD) or the median (Me) with the interquartile range (IQR). Categorical variables were expressed as frequencies and percentages. The Kruskal–Wallis test was used to compare continuous variables between patient subgroups. Whether the difference between subgroups was statistically significant was further tested by post hoc pairwise comparisons using pairwise Wilcoxon rank-sum tests with manual multiple-comparison correction (Bonferroni). Fisher’s exact test was used to compare ICU mortality between subgroups.

Associations between ROTEM parameters and ICU mortality were evaluated using multiple logistic regression. Odds ratios (ORs) with corresponding 95% confidence intervals (CIs) were calculated to quantify the strength and direction of associations. Given the high correlation among ROTEM parameters, key functional endpoints were pre-specified for mortality prediction: EXTEM CT for clot initiation, EXTEM AA and CFT for clot propagation, EXTEM A5 and MCF for overall clot firmness, FIBTEM A5 and MCF for clot firmness due to function of fibrinogen/factor XIII, ML for fibrinolysis, and difference in MCF between EXTEM and FIBTEM for PLT contribution to clot strength. The primary multivariable logistic regression analysis included the following confounders: age, sex, and SOFA score. Initially SOFA was named as a confounder; however, the SIC score itself incorporates SOFA, so keeping it in the mortality prediction model would introduce substantial mathematical coupling/overadjustment and was dropped; therefore, in the sensitivity analysis only age and sex were included. Every pre-specified key ROTEM parameter was assessed separately using multivariate logistic regression, taking into account the aforementioned confounders. A *p*-value < 0.05 was considered statistically significant.

Due to the retrospective nature of the study, the Bioethics Committee of the Medical University of Silesia decided that it does not require formal assessment.

## 3. Results

Data of all patients diagnosed with sepsis or septic shock in whom ROTEM (at minimum EXTEM and FIBTEM assays) was performed were retrieved (n = 206). The following groups of patients were excluded from the analysis: acute liver dysfunction (n = 29), therapeutic anticoagulation (n = 6), transfusion of cryoprecipitate and fresh frozen plasma (n = 2), and pregnancy (n = 2). Finally, data from 167 patients were analyzed.

The median age in the study group was 66.0 (IQR 55.0–72.0) years. There were 87 (52.1%) males in the study group. Six (3.6%) patients were receiving acetylsalicylic acid (ASA), whereas one (0.6%) was receiving a P2Y12 antiplatelet agent. Twenty-five (15.0%) patients were on prophylactic enoxaparin and 21 (12.6%) on prophylactic dalteparin before ROTEM testing. The median time interval between the last subcutaneous injection of low-molecular-weight heparin and blood withdrawal for ROTEM testing was 12.0 (IQR 12.0–19.0) hours. The median SOFA score was 6.0 (IQR 5.0–9.0) points, whereas the median SOFA without the PLT component was 6.0 (IQR 4.0–8.0) points. There were 90 (53.9%) patients diagnosed with septic shock. Sixty patients (35.9%) died during hospitalization in the ICU—37 (41.1%) among patients with septic shock and 23 (29.9%) in patients diagnosed with sepsis. Biochemical parameters in the study population are presented in Table 1.

The study group was characterized by high concentrations of inflammatory markers, exceeding the upper reference range multiple times—35.2 times for CRP, 29.3 times for IL-6, and 6.0 times for PCT. Mild kidney dysfunction was present in the study patients.

**Figure 1:**
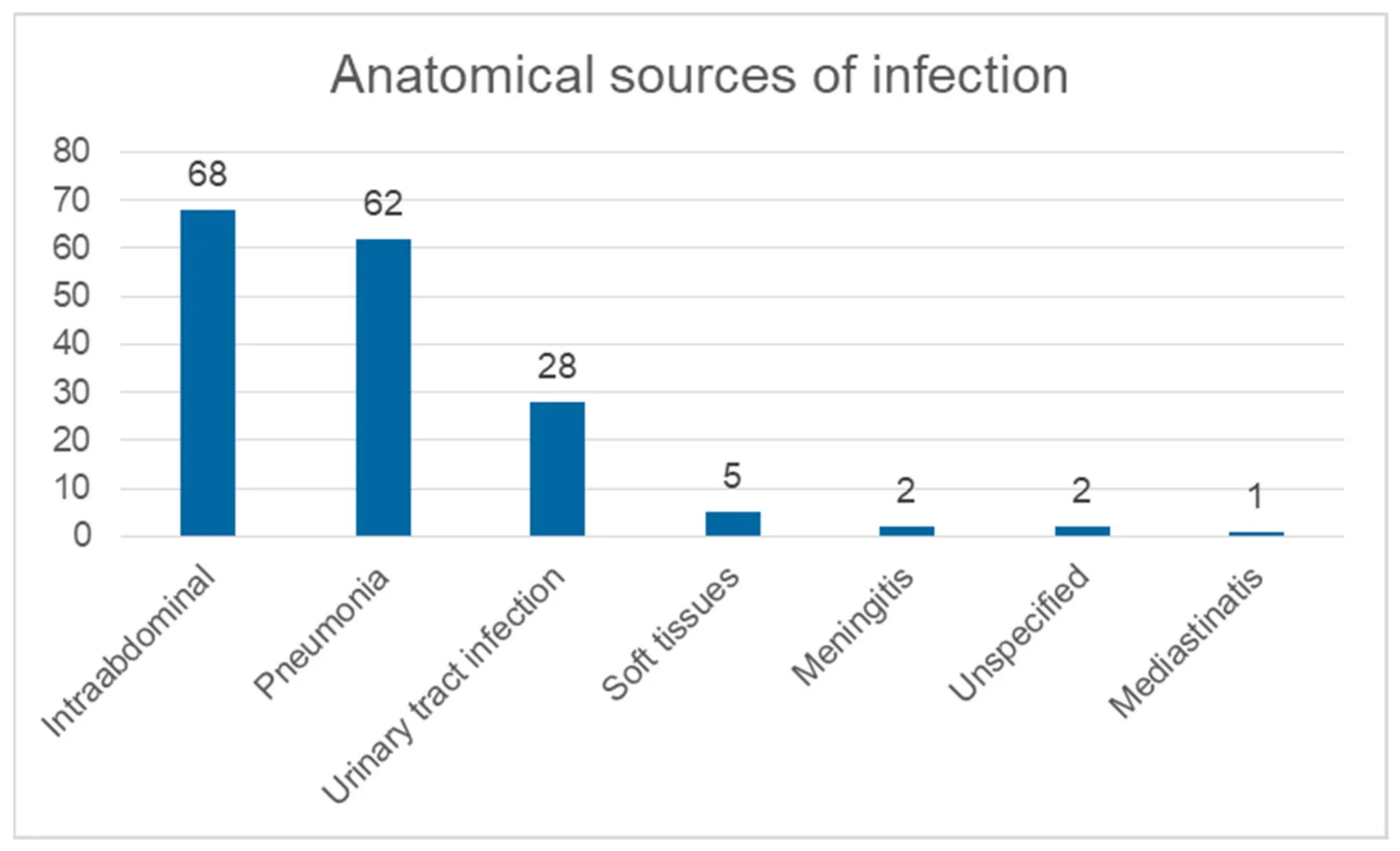
Anatomical sources of infection in the study population.

The anatomical sources of infection are presented in Figure 1.

There were 40 (23.9%) patients with SIC (subgroup SIC), 24 (14.4%) with sepsis-induced overt DIC (subgroup DIC), and 103 (61.7%) patients who did not present with either SIC or DIC (subgroup No-SIC-DIC). Among SIC patients, 28 (70.0%) had 4 points, 10 (25.0%) had 5 points, and two (5.0%) had 6 points on the ISTH SIC score. Among patients with DIC, 20 (83.3%) had 5 points, and four (16.7%) had 6 points on the ISTH overt-DIC score.

Logistic regression analysis showed that the coagulopathy subgroup was not a predictor of ICU mortality (OR = 0.97, 95% CI 0.61–1.56, *p* = 0.91), even after adjusting for age (*p* = 0.12), sex (*p* = 0.30), and SOFA (OR = 1.28, 95% CI 1.11–1.47, *p* < 0.01).

Standard coagulation parameters across study subgroups are presented in Table 2.

Fibrinogen concentration was above the upper reference range and did not differ significantly across the subgroups. There was a progressive increase in PT from No-SIC-DIC, through the SIC to DIC subgroup. The concentration of DD was severely increased in the DIC subgroup and moderately increased in the No-SIC-DIC and DIC subgroups. Platelet count was lower in both SIC and DIC compared to the No-SIC-DIC subgroup, corresponding to mild thrombocytopenia.

The INTEM parameters across the study subgroups are presented in Table 3.

The only INTEM parameters that were outside reference ranges were A10 and A20 in the No-SIC-DIC subgroup. Propagation of the clot (CFT, AA) was worse, and clot amplitudes at different time points were lower in both SIC and DIC compared to the No-SIC-DIC subgroup.

The EXTEM parameters across the study subgroups are presented in Table 4.

EXTEM CT was prolonged in both SIC and DIC compared to the No-SIC-DIC subgroup. Clot propagation (CFT, AA) was worse, and all clot amplitudes were lower in both SIC and DIC compared to the No-DIC-SIC subgroup. Fibrinolytic activity was lower in both SIC and DIC compared to the No-SIC-DIC subgroup, whereas in the DIC subgroup, fibrinolytic activity was low according to the literature [13].

In FIBTEM, early clot amplitudes were lower in SIC compared to the No-SIC-DIC subgroup (Table 5). There were no differences between SIC and DIC subgroups as far as any clot amplitude was concerned.

Although APTEM results were retrieved, they were not reported because fibrinolysis was uniformly low across the cohort, and APTEM tracings did not yield additional interpretive value beyond EXTEM and FIBTEM lysis indices.

Analysis of platelet contribution to clot strength (PLTEM), calculated as the difference in MCF between EXTEM and FIBTEM, showed various results across subgroups (*p* = 0.02), with a median of 42.0 (IQR 37.0–47.0) mm. Platelet contribution to clot was lower in the DIC subgroup (36, IQR 29.5–45.0 mm) compared to the SIC (42.0, IQR 37.0–47.5 mm) and No-SIC-DIC (43.0, IQR 39.0–47.0 mm) subgroups.

In the primary multivariable logistic regression model adjusted for age, sex, and SOFA score, several pre-specified ROTEM parameters were analyzed (Table 6). Higher FIBTEM clot amplitudes were associated with mortality.

In the multivariable logistic regression sensitivity model, without a problematic confounder (SOFA) included, none of the pre-specified ROTEM parameters was associated with ICU mortality in the presented cohort (Table 7).

## 4. Discussion

The present retrospective cohort is distinct from the previously published prospective study in early sepsis [5]. No patients or ROTEM measurements overlap between cohorts. The current analysis provides novel insights by comparing thromboelastometric profiles across ISTH-defined SIC and sepsis-induced overt DIC, using full ROTEM panels and multivariable mortality modeling, which were not addressed in the prior study. The present study showed several differences in ROTEM parameters between patients with SIC or sepsis-induced overt DIC and septic patients without coagulopathy. These differences pertained to every phase of the clot dynamics curve—initiation of coagulation, clot propagation, clot firmness, and fibrinolysis. Patients with sepsis-associated coagulopathy tended to have slower initiation of coagulation, poorer clot propagation, lower clot firmness, lower early platelet contribution to clot strength, and lower fibrinolytic activity compared to septic patients without coagulopathy. What is important is that there was only one ROTEM parameter that was significantly different between SIC and sepsis-induced overt DIC patients in the studied cohort, namely PLT contribution to clot strength. This aligns with the recent publication by Iba et al. who underscore the currently limited diagnostic utility of viscoelastic testing for detection of early hemostatic features of SIC [14]. The findings from the present study likely reflect that both SIC and DIC represent points along the same sepsis-driven coagulopathy continuum, characterized by inflammation-mediated thrombin generation, endothelial injury, and impaired fibrinolysis. Because ROTEM assesses functional clot dynamics rather than the laboratory variables used in SIC and DIC scoring systems, moderate differences in PT or fibrinogen may not translate into measurable differences in clot initiation or clot firmness. Furthermore, sepsis frequently produces a compensated viscoelastic profile with preserved clot firmness and minimal fibrinolysis, which may appear similar across SIC and overt DIC. Moreover, cohort characteristics may have reduced detectable separation. There were only four patients (all patients with score 6) with an ISTH overt DIC score > 5, which is the cut-off for diagnosing DIC, with a maximum score of 8 points. Limited representation of fulminant DIC phenotypes may have led to these indiscriminate thromboelastometric pictures.

Although fibrinolytic activity did not differ significantly between SIC and overt DIC in the present cohort, it is noteworthy that fibrinolysis in the overt DIC subgroup was low [15]. This finding is consistent with the characteristic hypofibrinolytic phenotype of sepsis-associated coagulopathy, driven largely by increased plasminogen activator inhibitor 1 (PAI-1) expression and impaired plasmin generation. As a result, patients meeting criteria for overt DIC may exhibit suppressed fibrinolysis on ROTEM, contributing to microvascular fibrin deposition and organ dysfunction.

Sepsis triggers complex, often competing, hemostatic processes. On the one hand, endothelial activation and thrombin generation promote fibrin deposition; on the other hand, consumption of coagulation factors and platelets can increase bleeding risk [16]. In the present study, ROTEM showed preserved coagulation and lack of hyperfibrinolysis. It is important to stress that, apart from minimally increased EXTEM CT, ROTEM parameters in patients with SIC and sepsis-induced overt DIC were within reference ranges. The overall preservation of clot firmness suggests that, despite meeting conventional laboratory criteria for SIC or overt-DIC, these patients exhibit a compensated viscoelastic profile typical of sepsis-associated coagulopathy. In such a phenotype, fibrinogen levels are often maintained or elevated, platelet function may be partially preserved, and fibrinolysis is frequently suppressed, resulting in ROTEM values that remain within normal limits. Administering procoagulant agents in this clinical setting could therefore be harmful, as it may exacerbate the underlying pro-thrombotic tendency and increase the risk of microvascular fibrin deposition and organ dysfunction.

The hypofibrinolytic profile and preserved fibrin-driven clot strength observed in the present cohort are consistent with contemporary models of sepsis-associated thromboinflammation and endothelial dysfunction. Sepsis induces a profound shift toward a procoagulant, antifibrinolytic state driven by endothelial injury, Weibel–Palade body release, PAI-1 upregulation, and impaired endogenous anticoagulant pathways, resulting in sustained fibrin deposition despite conventional laboratory evidence of coagulopathy. Recent mechanistic work highlights that infection-associated endothelial activation promotes a hypercoagulable, fibrin-rich phenotype with suppressed fibrinolysis, even in the presence of thrombocytopenia or prolonged PT/aPTT [17]. Similarly, infection-driven endothelial dysfunction has been shown to maintain clot firmness and limit fibrinolytic activity through enhanced thrombin generation and attenuated plasmin formation [18]. Within this framework, the preserved clot firmness and low fibrinolytic activity seen in both SIC and overt DIC subgroups likely reflect endothelial-mediated thromboinflammatory responses rather than discrete viscoelastic signatures of SIC versus DIC. These findings support the concept that viscoelastic profiles in sepsis often represent a compensated, fibrin-dominant phenotype shaped by endothelial pathology rather than isolated abnormalities in coagulation factor consumption.

The present study finally did not find any associations between pre-specified ROTEM parameters and ICU mortality after adjustment for age and sex in the sensitivity analysis. In the primary model adjusted for age, sex, and SOFA, only the FIBTEM parameters reflecting fibrin-based clot firmness (A5 and MCF) showed a significant association with ICU mortality, suggesting that increased fibrinogen-dependent clot firmness may indicate a more severe inflammatory–pro-thrombotic phenotype. However, in the sensitivity analysis without SOFA, none of the ROTEM parameters remained significant, indicating that the observed associations were dependent on model specification and were not robust across adjustment strategies. On the contrary, there were some publications that linked decreased fibrinolytic activity to mortality in septic patients [19].

The presented study has several limitations. The retrospective single-center design and exclusion of patients with liver failure or recent hemostatic therapy limit generalizability to broader septic populations. Rotational thromboelastometry is sensitive to pre-analytic and analytic variables such as sample timing relative to sepsis onset and therapies, reagent lots, and device calibration. The study did not standardize sampling timing; therefore, temporal heterogeneity could attenuate or exaggerate observed associations. The narrow distribution of overt DIC scores, with most patients at the diagnostic threshold of 5 points and only four patients scoring 6 points, substantially limited the ability to distinguish advanced DIC from SIC using ROTEM. The mechanically inferred platelet component is an indirect measure of platelet function; dedicated platelet function testing (e.g., aggregometry, PFA-100, flow cytometry) to validate PLTEM against biochemical or functional assays was not performed.

## 5. Conclusions

Rotational thromboelastometry shows distinct features in patients with SIC and sepsis-induced overt DIC compared with patients without coagulopathy. In the current cohort the only ROTEM parameter that distinguished overt DIC from SIC was PLT contribution to clot strength. No ROTEM parameter was associated with short-term mortality in the present cohort.

## Figures and Tables

**Table 1:** Biochemical parameters in the study population.

Parameter	Median Value, IQR ^1^	Reference Range
Interleukin 6 [pg mL^−1^] (59 obs.)	203.0 (76.3–928.0)	<7.0
Procalcitonin [ng mL^−1^] (161 obs.)	2.9 (0.9–18.0)	<0.5
C-reactive protein [mg L^−1^]	176.0 (102.0–272.0)	<5.0
Creatinine [mg dL^−1^]	1.2 (0.7–1.7)	0.51–0.95 (F ^2^)/0.67–1.17 (M ^3^)
Creatinine clearance [mL min^−1^]	63.0 (38.0–97.0)	85.0–125.0 (F)/75.0–115.0 (M)
MDRD ^4^ eGFR ^5^ [mL min^−1^ 1.73 m^−2^]	56.7 (34.9–60.0)	>60.0
CKD-EPI ^6^ eGRF [mL min^−1^ 1.73 m^−2^]	56.6 (31.2–94.4)	≥90.0
Blood urea nitrogen [mg dL^−1^]	29.8 (20.8–50.5)	7.90–20.0
Urea [mg dL^−1^]	63.8 (44.6–107.5)	7.0–20.0
Bilirubin [mg dL^−1^]	0.54 (0.37–0.85)	0.30–1.00
Aspartate aminotransferase [U L^−1^]	41.3 (26.5–105.0)	<40.0
Alanine aminotransferase [U L^−1^]	33.6 (18.2–69.7)	<40.0

^1^ Interquartile range. ^2^ Female. ^3^ Male. ^4^ Modification of Diet in Renal Disease. ^5^ Estimated glomerular filtration rate. ^6^ Chronic Kidney Disease-Epidemiology Collaboration.

**Table 2:** Standard coagulation parameters across study subgroups.

Parameter	No-SIC-DIC ^1^	SIC	DIC	*p*	Reference Range
Fibrinogen [mg dL^−1^]	535.0 (411.0–683.0)	455.5 (347.0–594.0)	546.0 (380.0–638.0)	0.28	200.0–393.0
**Prothrombin time [s]**	**13.2 (12.4–14.2)**	**16.6 (14.9–19.4)**	**20.2 (17.3–23.0)**	**<0.01**	**9.4–12.5**
**Prothrombin activity [%]**	**81.0 (73.0–92.0)**	**61.5 (47.5–69.5)**	**45.5 (38.0–55.5)**	**<0.01**	**80.0–120.0**
**INR ^2^**	**1.10 (1.03–1.20)**	**1.42 (1.23–1.66)**	**1.76 (1.45–1.93)**	**<0.01**	**0.80–1.20**
**aPTT ^3^ [s]**	**31.7 (29.3–35.5)**	36.3 (32.3–39.6)	36.2 (32.3–42.1)	**<0.01**	**25.4–36.9**
Thrombin time [s]	15.5 (14.6–16.9)	16.0 (15.1–18.4)	17.1 (15.0–18.7)	0.05	10.3–16.6
**D-dimers [ng mL^−1^]**	2874.0 (1543.0–5956.0)	2277.0 (1523.0–4624.0)	**6801.0 (5796.0–18,322.0)**	**<0.01**	**<500.0**
**Platelets [10^9^ L^−1^]**	**222.0 (176.0–297.0)**	129.0 (95.0–196.0)	112.5 (65.5–238.5)	**<0.01**	**130–400**

^1^ No-sepsis-induced coagulopathy-disseminated intravascular coagulation. ^2^ International normalized ratio. ^3^ Activated partial thromboplastin time. In Bold Statistically Significant Parameters.

**Table 3:** INTEM rotational thromboelastometry parameters across study subgroups (147 obs).

Parameter	No-SIC-DIC ^1^	SIC	DIC	*p*	Reference Range
Clotting time [s]	194.0 (179.0–212.0)	196.0 (186.5–215.0)	207.0 (183.0–239.0)	0.25	100.0–240.0
**Clot formation time [s]**	**56.0 (48.0–67.0)**	68.5 (56.0–85.0)	70.0 (53.0–90.0)	**<0.01**	**30.0–110.0**
**Alfa angle [°]**	**79.0 (76.0–80.0)**	76.5 (74.0–78.5)	76.0 (74.0–79.0)	**0.01**	**70.0–83.0**
**Amplitude at 5 min. [mm]**	**58.0 (52.0–63.0)**	49.5 (42.5–55.0)	51.0 (40.0–59.0)	**<0.01**	**-**
**Amplitude at 10 min. [mm]**	**67.0 (62.0–71.0)**	60.5 (54.0–65.0)	60.5 (51.0–67.0)	**<0.01**	**44.0–66.0**
**Amplitude at 20 min. [mm]**	**72.0 (67.0–75.0)**	66.0 (60.0–70.0)	65.0 (58.0–71.0)	**<0.01**	**50.0–71.0**
**Maximal clot amplitude [mm]**	**72.0 (67.0–75.0)**	66.0 (61.5–72.0)	67.0 (59.0–71.0)	**<0.01**	**50.0–72.0**
Maximal lysis [%]	5.0 (2.0–8.0)	3.0 (1.0–6.5)	3.0 (1.0–7.0)	0.10	0.0–15.0
Lysis index at 30 min. [%]	100.0 (99.0–100.0)	100.0 (99.0–100.0)	100.0 (99.0–100.0)	0.81	94.0–100.0
Lysis index at 45 min. [%]	97.0 (95.0–99.0)	99.0 (95.0–100.0)	98.0 (96.0–100.0)	0.23	-

^1^ No-sepsis-induced coagulopathy-disseminated intravascular coagulation. In Bold Statistically Significant Parameters.

**Table 4:** EXTEM rotational thromboelastometry parameters across study subgroups (167 obs).

Parameter	No-SIC-DIC ^1^	SIC	DIC	*p*	Reference Range
**Clotting time [s]**	**76.0 (70.0–86.0)**	91.0 (76.5–97.5)	97.5 (81.5–111.0)	**<0.01**	**38.0–79.0**
**Clot formation time [s]**	**57.0 (48.0–67.0)**	71.5 (62.0–90.0)	74.5 (53.5–100.5)	**<0.01**	**34.0–159.0**
**Alfa angle [°]**	**79.0 (77.0–80.0)**	76.0 (73.0–78.5)	77.0 (75.5–80.0)	**<0.01**	**63.0–83.0**
**Amplitude at 5 min. [mm]**	**59.0 (52.0–64.0)**	49.0 (42.5–55.5)	47.5 (35.0–59.0)	**<0.01**	**-**
**Amplitude at 10 min. [mm]**	**68.0 (62.0–72.0)**	59.5 (54.0–66.0)	58.0 (45.5–67.5)	**<0.01**	**43.0–65.0**
**Amplitude at 20 min. [mm]**	**71.0 (66.0–75.0)**	65.0 (60.0–70.5)	64.5 (53.0–71.0)	**<0.01**	**50.0–71.0**
**Maximal clot amplitude [mm]**	**72.0 (68.0–75.0)**	66.0 (62.5–71.0)	66.5 (56.5–71.5)	**<0.01**	**50.0–72.0**
**Maximal lysis [%]**	**7.0 (4.0–16.0)**	4.5 (1.5–8.5)	2.5 (0.5–8.0)	**<0.01**	**0.0–15.0**
**Lysis index at 30 min. [%]**	**100.0 (98.0–100.0)**	100.0 (100.0–100.0)	100.0 (99.0–100.0)	**<0.01**	**94.0–100.0**
**Lysis index at 45 min. [%]**	**97.0 (91.0–99.0)**	98.0 (95.0–100.0)	99.0 (95.0–100.0)	**<0.01**	

^1^ No-sepsis-induced coagulopathy-disseminated intravascular coagulation. In Bold Statistically Significant Parameters.

**Table 5:** FIBTEM rotational thromboelastometry parameters across study subgroups (167 obs).

Parameter	No-SIC-DIC ^1^	SIC	DIC	*p*	Reference Range
**Clotting time [s]**	**73.0 (65.0–79.0)**	80.0 (65.5–89.5)	87.5 (75.5–99.5)	**<0.01**	**38.0–62.0**
**Clot formation time [s]**	**88.0 (58.0–173.0)**	157.0 (67.0–518.0)	190.5 (77.0–540.0)	**0.03**	**-**
**Alfa angle [°]**	**78.0 (75.0–79.0)**	75.0 (69.5–78.0)	76.0 (70.0–79.0)	**0.04**	**-**
**Amplitude at 5 min. [mm]**	**23.0 (18.0–29.0)**	**19.5 (14.0–26.5)**	21.0 (18.0–26.0)	**0.03**	
**Amplitude at 10 min. [mm]**	**26.0 (21.0–32.0)**	**21.0 (15.5–28.0)**	23.0 (19.5–28.5)	**0.02**	**7.0–23.0**
**Amplitude at 20 min. [mm]**	**27.0 (22.0–34.0)**	**22.5 (17.0–30.0)**	25.0 (22.5–31.0)	**0.04**	**8.0–24.0**
Maximal clot amplitude [mm]	29.0 (23.0–36.0)	23.5 (17.5–32.5)	27.0 (25.0–32.5)	0.06	9.0–25.0
Maximal lysis [%]	0.0 (0.0–1.0)	0.0 (0.0–0.5)	0.0 (0.0–1.0)	0.82	**-**
Lysis index at 30 min. [%]	100.0 (100.0–100.0)	100.0 (100.0–100.0)	100.0 (100.0–100.0)	0.49	**-**
Lysis index at 45 min. [%]	100.0 (100.0–100.0)	100.0 (100.0–100.0)	100.0 (100.0–100.0)	0.61	**-**

^1^ No-sepsis-induced coagulopathy-disseminated intravascular coagulation. In Bold Statistically Significant Parameters.

**Table 6:** Primary multiple logistic regression analysis of pre-specified ROTEM parameters for intensive care unit mortality prediction adjusted for age, sex, and SOFA score.

Parameter	Odds Ratio (95% CI ^1^)	*p*
EXTEM ^2^ clotting time	1.01 (0.99–1.02)	0.26
EXTEM clot formation time	1.00 (0.98–1.01)	0.50
EXTEM alpha angle	1.02 (0.94–1.12)	0.59
EXTEM clot amplitude at 5 min. (early firmness)	1.02 (0.98–1.05)	0.33
EXTEM maximal clot firmness (late firmness)	1.02 (0.98–1.08)	0.32
EXTEM maximal lysis	1.01 (0.98–1.03)	0.61
**FIBTEM ^3^ clot amplitude at 5 min. (early firmness)**	**1.06 (1.01–1.11)**	**0.02**
**FIBTEM maximal clot firmness (late firmness)**	**1.04 (1.00–1.09)**	**0.03**
EXTEM-FIBTEM maximal clot firmness	0.97 (0.92–1.01)	0.12

^1^ Confidence interval. ^2^ Extrinsic coagulation pathway assay. ^3^ Fibrinogen/factor XIII assay. In Bold Statistically Significant Parameters.

**Table 7:** Multiple logistic regression sensitivity analysis of pre-specified ROTEM parameters for intensive care unit mortality prediction, adjusted for age and sex.

Parameter	Odds Ratio (95% CI ^1^)	*p*
EXTEM ^2^ clotting time	1.01 (0.99–1.02)	0.20
EXTEM clot formation time	1.00 (0.99–1.01)	0.57
EXTEM alpha angle	0.98 (0.91–1.05)	0.53
EXTEM clot amplitude at 5 min. (early firmness)	1.00 (0.97–1.03)	0.97
EXTEM maximal clot firmness (late firmness)	1.01 (0.97–1.06)	0.59
EXTEM maximal lysis	1.00 (0.97–1.02)	0.73
FIBTEM ^3^ clot amplitude at 5 min. (early firmness)	1.04 (0.99–1.08)	0.07
FIBTEM maximal clot firmness (late firmness)	1.03 (0.99–1.07)	0.09
EXTEM-FIBTEM maximal clot firmness	0.97 (0.93–1.01)	0.13

^1^ Confidence interval. ^2^ Extrinsic coagulation pathway assay. ^3^ Fibrinogen/factor XIII assay.

## Data Availability

The data presented in this study are available on request from the corresponding author.

## References

[1] Rudd K.E., Johnson S.C., Agesa K.M., Shackelford K.A., Tsoi D., Kievlan D.R., Colombara D.V., Ikuta K.S., Kissoon N., Finfer S. (2020). Global, regional, and national sepsis incidence and mortality, 1990–2017: Analysis for the Global Burden of Disease Study. Lancet.

[2] Iba T., Levy J.H., Warkentin T.E., Thachil J., van der Poll T., Levi M., the Scientific and Standardization Committee on DIC, and the Scientific and Standardization Committee on Perioperative and Critical Care of the International Society on Thrombosis and Haemostasis (2019). Diagnosis and management of sepsis-induced coagulopathy and disseminated intravascular coagulation. J. Thromb. Haemost..

[3] Hartmann J., Hermelin D., Levy L.H. (2022). Viscoelastic testing: An illustrated review of technology and clinical applications. Res. Pract. Thromb. Haemost..

[4] Forster E.K., Hendel S., Mitra B. (2022). Detection of acute traumatic coagulopathy by viscoelastic haemostatic assays compared to standard laboratory tests: A systematic review. Transfus. Med. Hemotherapy.

[5] Czempik P.F., Wiórek A. (2024). Rotational thromboelastometric profile in early sepsis: A prospective cohort study. Biomedicines.

[6] Hanh-Duyen B.T., Kien T.G., Khoi L.M. (2023). Coagulation profiles in patients with sepsis/septic shock identify mixed hypo-hypercoagulation patterns based on rotational thromboelastometry: A prospective observational study. Thromb. Res..

[7] Słomka A., Kowalewski M., Żekanowska E. (2021). Hemostasis in coronavirus disease 2019-lesson from viscoelastic methods: A systematic review. Thromb. Haemost..

[8] Singer M., Deutschman C.S., Seymour C.W., Shankar-Hari M., Annane D., Bauer M., Bellomo R., Bernard G.R., Chiche J.-D., Coopersmith C.M. (2016). The third international consensus definitions for sepsis and septic shock (Sepsis-3). JAMA.

[9] Vincent J.L., Moreno R., Takala J., Willatts S., De Mendonça A., Bruining H., Reinhart C.K., Suter P.M., Thijs L.G. (1996). The SOFA (Sepsis-related Organ Failure Assessment) score to describe organ dysfunction/failure. On behalf of the Working Group on Sepsis-Related Problems of the European Society of Intensive Care Medicine. Intensive Care Med..

[10] Iba T., Nisio M.D., Levy J.H., Kitamura N., Thachil J. (2017). New criteria for sepsis-induced coagulopathy (SIC) following the revised sepsis definition: A retrospective analysis of a nationwide survey. BMJ Open.

[11] Taylor F.B., Toh C.H., Hoots W.K., Wada H., Levi M., the Scientific Subcommittee on Disseminated Intravascular Coagulation (DIC) of the International Society on Thrombosis and Haemostasis (ISTH) (2001). Towards definition, clinical and laboratory criteria, and a scoring system for disseminated intravascular coagulation. Thromb. Haemost..

[12] Iba T., Levy J.H., Yamakawa K., Thachil J., Warkentin T.E., Levi M., the Scientific and Standardization Committee on DIC of the International Society on Thrombosis and Haemostasis (2019). Proposal of a two-step process for the diagnosis of sepsis-induced disseminated intravascular coagulation. J. Thromb. Haemost..

[13] Iba T., Levy J.H., Maier C.L., Helms J., Umemura Y., Moore H., Othman M., Thachil J., Connors J.M., Levi M. (2025). Updated definition and scoring of disseminated intravascular coagulation in 2025: Communication from the ISTH SSC Subcommittee on Disseminated Intravascular Coagulation. J. Thromb. Haemost..

[14] Iba T., Helms J., Levy J.H. (2025). The role of viscoelastic tests in the diagnosis of sepsis-induced coagulopathy (SIC). Ann. Intensive Care.

[15] Belfiore J., Castellani Nicolini N., Bindi M.L., Saner F.H., Blasi A., Piaggi P., Ghinolfi D., Biancofiore G. (2026). Evaluation of low fibrinolytic activity by rotational thromboelastometry and outcomes in liver transplantation: A single-center prospective study. Liver Transplant..

[16] Girardis M., David S., Ferrer R., Helms J., Juffermans N.P., Martin-Loeches I., Povoa P., Russell L., Shankar-Hari M., Iba T. (2024). Understanding, assessing and treating immune, endothelial and haemostasis dysfunctions in bacterial sepsis. Intensive Care Med..

[17] Pamporis K., Karakasis P., Pantelidaki A., Goutis P.A., Grigoriou K., Theofilis P., Katsaouni A., Botis M., Karanikola A.-E., Milaras N. (2025). Sepsis-induced cardiomyopathy and cardiac arrhythmias: Pathophysiology and implications for novel therapeutic approaches. Biomedicines.

[18] Karakasis P., Nasoufidou A., Sagris M., Fragakis N., Tsioufis K. (2024). Vascular alterations following COVID-19 infection: A comprehensive literature review. Life.

[19] Iba T., Levi M., Levy J.H. (2020). Sepsis-induced coagulopathy and disseminated intravascular coagulation. Semin. Thromb. Hemost..

